# Influence of Pennation Angle and Muscle Thickness on Mechanomyographic Amplitude–Torque Relationships and Sex-Related Differences in the Vastus Lateralis

**DOI:** 10.3390/jfmk8020053

**Published:** 2023-05-02

**Authors:** Michael Trevino, Sergio Perez, Stephanie Sontag, Alex Olmos, Sunggun Jeon, Lyric Richardson

**Affiliations:** Applied Neuromuscular Physiology Laboratory, Department of Kinesiology, Applied Health, and Recreation, Oklahoma State University, Stillwater, OK 74078, USA

**Keywords:** females, log transform, males, mechanomyography, muscle thickness, pennation angle, vastus lateralis

## Abstract

This study examined potential sex-related differences and correlations among the pennation angle (PA), muscle thickness (MT), and mechanomyographic amplitude (MMG_RMS_)–torque relationships of the vastus lateralis (VL) in 11 healthy males and 12 healthy females. The PA and MT of the VL were quantified with ultrasound. Participants performed an isometric muscle action of the knee extensors that linearly increased to 70% of maximal strength followed by a 12 s plateau. MMG was recorded from the VL. Linear regression models were fit to the log-transformed MMG_RMS_–torque relationships to calculate *b* terms (slopes) for the linearly increasing segment. MMG_RMS_ was averaged during the plateau. Males exhibited greater PA (*p* < 0.001), MT (*p* = 0.027), *b* terms (*p* = 0.005), and MMG_RMS_ (*p* = 0.016). The *b* terms were strongly (*p* < 0.001, *r* = 0.772) and moderately correlated (*p* = 0.004, *r* = 0.571) with PA and MT, respectively, while MMG_RMS_ was moderately correlated with PA (*p* = 0.018, *r* = 0.500) and MT (*p* = 0.014, *r* = 0.515). The greater mechanical behavior of individuals possessing a larger PA and MT of the VL may reflect increased cross-bridge activity within the muscle fibers. Additionally, PA may help explain sex-related differences in MMG_RMS_ between sexes.

## 1. Introduction

Ultrasound imaging is commonly used to quantify human muscle architecture [1,2,3]. Measures such as pennation angle and muscle thickness are frequently examined due to their influence on muscle strength [3,4,5,6]. For example, a muscle with larger pennation angles can have more fibers arranged in parallel for a respective volume, consequently allowing greater force-generating capabilities [7]. In addition, there are numerous reports that maximal strength is positively related to muscle size [4,8,9,10]. Therefore, pennation angle and muscle thickness are commonly of interest as they are key determinants for muscle performance.

The influence of pennation angle and muscle thickness on muscle activity has been frequently assessed during maximal and submaximal efforts [11,12,13,14]. Commonly, electromyography (EMG) is concurrently recorded to provide additional insight regarding skeletal muscle [15,16,17]. For example, relationships have been reported between changes in EMG amplitude (EMG_RMS_) and muscle thickness for the transverse abdominis [18], pennation angle and normalized EMG_RMS_ for the plantar flexors and dorsiflexors during isometric contractions ranging from low to high intensities [15], and between muscle thickness and normalized EMG_RMS_ for the rectus femoris during isometric contractions from low to maximal effort [19]. Therefore, evidence exists that the electrical activity of the muscle may be influenced by muscular architecture during maximal and submaximal contractions. However, markedly less is known regarding relationships between muscle architecture and the mechanical activity of muscle.

Surface mechanomyography (MMG) records low-frequency oscillations produced by muscle fibers during contractions [20]. It has been stated that MMG reflects the mechanical activity of neural activation [21]. For example, the amplitude of the MMG signal (MMG_RMS_) can be affected by active muscle stiffness resulting from the recruitment of motor units and their respective firing rates [22]. However, it appears that MMG_RMS_ can also be influenced by the physical properties of the motor unit pool. For example, greater *b* terms (slopes) calculated from the log-transformed MMG_RMS_–force/torque relationships during linearly increasing and decreasing muscle actions, and MMG_RMS_ values at steady force/torque, have been reported for individuals expressing greater amounts of type II percent myosin heavy chain isoform content [23,24,25,26]. In addition, MMG_RMS_–torque relationships can also be influenced by muscle architecture [27]. Sontag et al. [27] reported relationships between the muscle cross-sectional area of the vastus lateralis and the *b* terms (slopes) calculated from the log-transformed MMG_RMS_–torque relationships during an isometric ramp muscle action up to 70% of maximal strength, such as individuals with larger muscles exhibited *b* terms that plateaued later (greater slope values) throughout the torque spectrum. Due to previously reported positive relationships among muscle cross-sectional area, the relative increase in motor unit sizes with increments in recruitment thresholds, and type II percent myosin heavy chain expression for the vastus lateralis [28], Sontag et al. [27] suggested that individuals possessing greater muscle cross-sectional areas may have greater type II fiber diameters. Despite the association between muscle cross-sectional area and MMG_RMS_, no other studies have examined potential relationships among other muscle architecture measurements, such as muscle thickness and MMG.

Previously, Kawakami et al. [29] reported positive relationships (*r* = 0.61) between muscle thickness and pennation angle for the VL. It has been suggested that greater pennation angles allow for an increase in the packing of contractile proteins of the muscle [7]. Theoretically, this should allow greater cross-bridge activity within the muscle fibers, and, consequently, larger muscles would produce a greater amount of oscillations during contractions. Therefore, it is possible that relationships may also exist between pennation angle of the VL and MMG_RMS_. Similar to muscle thickness, relationships between pennation angle and MMG_RMS_–torque relationships have not been examined and also warrant investigation. In addition to relationships between muscle cross-sectional area and MMG_RMS_–torque relationships, Sontag et al. [27] reported sex-related differences in these variables. Consequently, muscle thickness and pennation angle may also help explain sex-related differences in the mechanical behavior of the VL.

Examining MMG_RMS_–torque relationships during linearly increasing muscle actions can become difficult due to the relatively large variability among individuals [30]. Thus, it has been suggested that the relationships should be examined on a subject-by-subject basis [31,32,33] since using ANOVA models or polynomial regression to analyze the composite MMG_RMS_ means at various percentages of maximal strength does not always accurately describe the individual patterns of response [30]. Consequently, researchers have applied natural log transformations to the MMG_RMS_ and torque values for each contraction in order to calculate slope values (*b* terms) and examine the individual patterns [25,34,35]. The 95% confidence intervals calculated around the group means for the *b* terms provide additional information regarding the linearity of the original MMG_RMS_–torque patterns. For example, the relationship is linear if the 95% confidence intervals include 1. In addition, 95% intervals that are less than 1 but greater than 0 indicate a nonlinear relationship with downward deceleration, as the rate of change is greater for torque than MMG_RMS_. During an isometric ramp muscle action up to 70% of maximal strength, positive relationships have been observed between muscle cross-sectional area and the *b* terms calculated from the log-transformed MMG_RMS_–torque relationships [27]. In addition, the *b* terms successfully differentiated the mechanical behavior of the VL between males and females during an isometric ramp up muscle action up to 70% of maximal strength [27]. Therefore, the purpose of this study was to measure muscle thickness, pennation angle, the *b* terms (slopes) calculated from the log-transformed MMG_RMS_–torque relationships during a linearly increasing contraction up to 70% maximal voluntary contraction (MVC), and MMG_RMS_ at 70% MVC steady torque of the VL for males and females. A trajectory that contains an increase to the targeted torque followed by a plateau was selected because ramp and step contractions are commonly used to investigate MMG_RMS_–torque/force relationships [21,22]. It was hypothesized that sex-related differences would exist for all variables, and muscle thickness and pennation angle would be correlated with MMG_RMS_ parameters.

## 2. Materials and Methods

### 2.1. Subjects

A total of 11 healthy males (means ± SD; age: 20.18 ± 2.04 years; height: 178.51 ± 4.28 cm; body mass: 78.63 ± 9.11 kg) and 12 healthy females (age: 21.33 ± 3.00 years; height: 164.58 ± 6.60 cm; body mass: 60.38 ± 10.24 kg) participated in this investigation. The subjects did not engage in any form of structured exercise in the 3 years prior to this investigation or report any previous or ongoing neuromuscular diseases or musculoskeletal injuries. Approval was granted from the university institutional review board for human subjects’ research before data collection commenced, and all participants signed an informed consent form prior to participating.

### 2.2. Ultrasound Imaging

Real-time brightness-mode (B-mode) ultrasonography (LOGIQ e, GE Healthcare, Chicago, IL, USA) was used to measure pennation angle, muscle thickness, and subcutaneous (sFAT) of the VL. Before ultrasound imaging, participants laid supine for 10 min to allow fluid redistribution and minimize measurement error [36,37]. The ultrasound scans of the VL were taken at the midpoint of the anterior superior iliac spine and the superior border of the patella on the right leg (similar position to MMG sensor). Water-soluble transmission gel was applied generously to the multi-frequency linear array probe (12 L-RS; 5–13 MHz; 38.4-mm field of view) to enhance acoustic coupling and reduce possible near-field artifacts. The probe was orientated longitudinally and parallel to the fascicular path in the sagittal plane, and it was determined that the probe alignment was appropriate when multiple fascicles were easily delineated without interruption across the image. All ultrasound images were analyzed with ImageJ software (version 1.46r; National Institutes of Health, Bethesda, MD, USA), and each image was scaled from pixels to cm using the straight-line function. Pennation angle was calculated as the angle between the muscle fascicle and the deep aponeurosis. The average for three pennation angle measurements was used in subsequent analyses. Muscle thickness was defined as the perpendicular distance between the superficial and deep aponeurosis. sFAT was quantified as the perpendicular distance between the skin and superficial aponeurosis of the muscle. It has been reported that sFAT can low-pass-filter the MMG signal [38,39]. Therefore, sFAT was quantified to examine whether relationships existed with the MMG_RMS_ parameters. Nonsignificant relationships would provide further confidence that sFAT was not a confounding factor for any MMG_RMS_ findings.

### 2.3. Isometric Strength Testing

Each subject was seated with restraining straps over the pelvis, trunk, and contralateral thigh, and the lateral condyle of the femur was aligned with the input axis of a Bidoex System 3 isokinetic dynamometer (Biodex Medical Systems, Shirley, NY, USA) in accordance with the Biodex user guide. (Biodex Pro Manual, Applications/Operations, 1998). All isometric knee extensor strength assessments were performed on the right leg at a flexion of 90°. Isometric strength of the right knee extensor muscles was measured using the torque signal from the Biodex System 3 isokinetic dynamometer.

**Figure 1:**
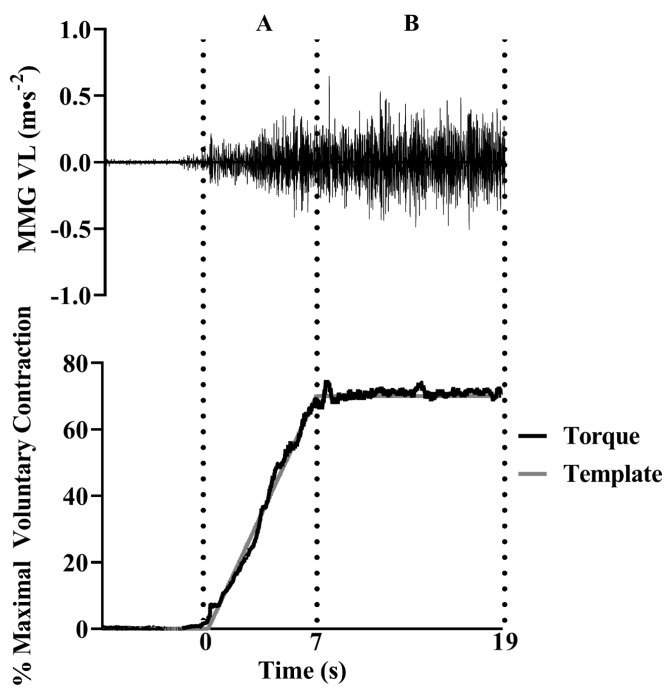
An example of the mechanomyographic (MMG) signal from the vastus lateralis (VL) during the 70% isometric muscle action for 1 participant (top). The torque (bottom) is overlaid on the targeted template as it appeared for the participant during the trial. The dashed lines represent the linearly increasing (**A**) and steady torque (**B**) segments of the template. The portion of the MMG signal that corresponded with the respective segment (**A**,**B**) was selected for analysis.

During experimental testing, subjects performed three three-second MVCs of the knee extensors with strong verbal encouragement. Three minutes of rest was given between trials, and the highest torque output was designated as the MVC. Following the MVCs, subjects performed an isometric submaximal muscle action. The template for the muscle action included a linearly increasing segment up to 70% MVC that increased 10% MVC/s to the target torque and a 12 s plateau (Figure 1). Therefore, the muscle action lasted 19 s. Subjects were digitally presented their torque output in real time on a monitor and were instructed to match the template as closely as possible. If they were not within 5% of the targeted template during the initial trial, they were given a second attempt after a 5-min rest period [40,41].

### 2.4. Mechanomyographic and Torque Signal Processing

During the submaximal muscle action, surface MMG signals were recorded from the VL with active miniature accelerometers (model 352A24, bandwidth = 10–8000 Hz, sensitivity of 98.8 mV m/s^2^, PCB Piezotronics, Inc, Depaw, NY, USA). The sensor was placed over the VL on the lateral/anterior portion of the muscle at half the distance of the greater trochanter and lateral condyle of the femur. Before sensor placement, the skin was shaved and cleaned with an alcohol wipe. The accelerometer was affixed to the skin with double-sided tape.

The MMG (m/s^2^) and torque (Nm) signals were simultaneously sampled at 2 kHz with a National Instruments compact data acquisition system (NI cDAQ-9174). The signals were bandpass-filtered (fourth-order Butterworth) at 5–100 Hz. For the submaximal muscle action, the torque and MMG signals were analyzed with consecutive, non-overlapping 0.25 s epochs [25,26,27,34,35]. The amplitude of the MMG signal was calculated with root mean square (RMS). The recorded signals were stored on a desktop computer and processed offline with custom-written software (LabVIEW, version 18; National Instruments, Austin, TX, USA).

### 2.5. Statistical Analysis

For the linearly increasing segment of the targeted torque template, the torque and MMG_RMS_ values for each quarter-second epoch underwent a natural log transformation, and simple linear regression models were applied [25,26,27,34,35]. The equations were represented as:

ln[*Y*] = *b*(ln[*X*]) + ln[*a*] (1)

where ln[*Y*] represents the natural log of the MMG_RMS_ values, ln[*X*] represents the natural log of the torque values, *b* represents the slope, and ln[*a*] = the natural log of the *y*-intercept. After the antilog transformation, this can also be expressed as an exponential equation:

*Y* = *aX^b^* (2)

where *Y* = the predicted MMG_RMS_ values, *X* represents torque, *b* represents the slope of Equation (1), and a represents the antilog of the y-intercept from Equation (1). For the 12 s steady torque segment, MMG_RMS_ was calculated by averaging the values during the entire 12 s targeted contraction torque [25,26,34,35]. The *b* terms were calculated with Microsoft Excel^®^ version 16.

Homogeneity of variances was assessed by performing preliminary Levene’s tests for all variables. All variables exhibited homogeneity (*p* > 0.081–0.975). Therefore, five separate independent sample *t*-tests were used to examine sex-related differences in *b* terms, MMG_RMS_ at steady torque, pennation angle, muscle thickness, and sFAT. Additionally, six Pearson’s product moment correlation coefficients were calculated comparing the *b* terms and MMG_RMS_ with pennation angle, muscle thickness, and sFAT. The level of significance was set at *p* ≤ 0.05. SPSS 25 was used to perform all statistical analyses (IBM Corporation, Armonk, NY, USA).

## 3. Results

### 3.1. Sex-Related Comparisons

**Figure 2:**
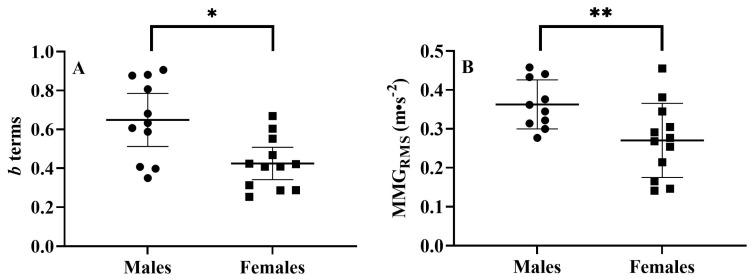
Left (**A**): plotted individual values and means ± 95% confidence intervals for *b* terms from the mechanomyographic amplitude (MMG_RMS_) vs. torque relationship of the vastus lateralis (VL) for the males and females. Right (**B**): plotted individual values and means ± SD for MMG_RMS_ of the VL during steady torque for the males and females. * Indicates greater *b* terms for the males than females (*p* = 0.005); ** indicates greater MMG_RMS_ for males than females (*p* = 0.016).

**Figure 3:**
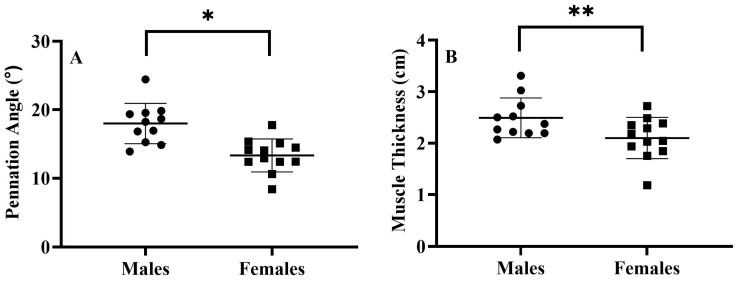
Plotted individual values and means ± SD for pennation angle (left graph (**A**)) and muscle thickness (right graph (**B**)) of the vastus lateralis for males and females. * Indicates greater pennation angle for the males than females (*p* < 0.001); ** indicates greater muscle thickness for males than females (*p* = 0.027).

Males exhibited greater *b* terms (*p* = 0.005, males = 0.649 ± 0.204, females = 0.424 ± 0.132, (Figure 2)); MMG_RMS_ at steady torque (*p* = 0.016, males = 0.36 ± 0.06 m/s^2^, females = 0.27 ± 0.10 m/s^2^ (Figure 2)); pennation angle (*p* < 0.001, males = 17.98 ± 2.94°, females = 13.33 ± 2.41° (Figure 3)); and muscle thickness (*p* = 0.027, males = 2.49 ± 0.39 cm, females = 2.10 ± 0.40 cm (Figure 3)) for the VL. Additionally, females possessed greater sFAT than males (*p* < 0.001, females = 1.43 ± 0.46 cm, males = 0.62 ± 0.27 cm).

### 3.2. Correlations

**Figure 4:**
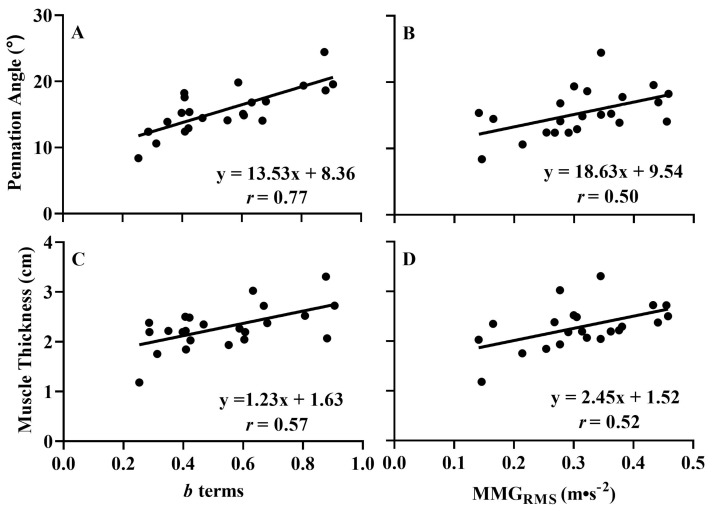
Left graphs: the plotted relationships between the *b* terms from the log-transformed mechanomyographic amplitude (MMG_RMS_)–torque relationships during the linearly increasing segment and pennation angle (**A**) and muscle thickness (**C**). Right graphs: the plotted relationships between MMG_RMS_ during steady torque and pennation angle (**B**) and muscle thickness (**D**).

The *b* terms were significantly correlated with pennation angle (*p* < 0.001, *r* = 0.772) and muscle thickness (*p* = 0.004, *r* = 0.571) (Figure 4) but not with sFAT (*p* = 0.263). Additionally, MMG_RMS_ at steady torque was significantly correlated with pennation angle (*p* = 0.018, *r* = 0.500) and muscle thickness (*p* = 0.014, *r* = 0.515) but not with sFAT (*p* = 0.154) (Figure 4). It has been reported that sFAT may have a low-pass filtering effect on the MMG signal [38,39]; however, the lack of relationships between sFAT and the *b* terms during the linearly increasing segment and MMG_RMS_ at steady torque suggests it was not responsible for the correlations among pennation angle and muscle thickness with MMG_RMS_ parameters.

### 3.3. Sequential Multiple Regression Models for Pennation Angle and Muscle Thickness

Due to the significant correlations, a secondary analysis was performed examining the possibility of predicting pennation angle and muscle thickness with MMG_RMS_ parameters. Preliminary analyses with Kolmogorov–Smirnov and Shapiro–Wilk tests confirmed (*p* > 0.05) normality and homoscedasticity for both the *b* terms and MMG_RMS_. Subsequently, the *b* terms and MMG_RMS_ were both entered individually and in combination by order of significance into separate linear regression models to investigate the potential for estimating pennation angle and muscle thickness, respectively. The tolerance and variable inflation factor for the *b* terms and MMG_RMS_ for the pennation angle and muscle thickness estimation models were >0.240 and <4.2, respectively. Therefore, collinearity was not indicated between variables [42]. The *b* terms were the initial independent variable entered into both estimation models, as they had the strongest relationship with pennation angle and muscle thickness. However, adding MMG_RMS_ to each model did not increase the ability to estimate pennation angle (*p* = 0.176) or muscle thickness (*p* = 0.099) greater than the *b* terms alone.

## 4. Discussion

As previously reported, males exhibited greater muscle thickness [43], pennation angles [43,44], and *b* terms for the VL [27]. Significant and novel findings include that pennation angle and muscle thickness accounted for large portions of the mechanical behavior of the VL during a linearly increasing muscle action up to and at a high-intensity targeted torque, and sex-related differences existed for MMG_RMS_ recorded from the VL at steady torque. Subsequently, muscle architecture was able to show differences in the mechanical behavior of the VL between males and females during linearly increasing and steady-state torque production. In addition, the MMG signal recorded during a high-intensity contraction may provide insight into the muscle architecture of the VL.

For the log-transformed MMG_RMS_–torque relationships during the linearly increasing segment of the targeted torque trajectory, all 23 relationships (100%) were significant. The 95% confidence interval range for the *b* terms (0.449–0.614) was greater than 0 yet less than 1, indicating that the relationships decelerated (plateaued) as torque increased at a greater rate than MMG_RMS_.

Previously, the *b* terms differentiated individuals as a function of the muscle cross-sectional area of the VL [27]. It was suggested that the positive relationships were the result of individuals with larger muscle cross-sectional area values possessing larger sized fibers that expressed type II characteristics, since males and females have a similar fiber type distribution (ratio of slow- and fast-twitch fibers) for the VL [45,46,47,48], and type II fibers possess greater force twitches [49] and are less likely to fuse in response to increasing excitation from the central nervous system [50]. Therefore, it was hypothesized that positive relationships would exist between muscle thickness and MMG_RMS_ parameters during the linearly increasing segment and at steady torque, which was supported in the current study. However, it should be noted that the relationships during the linearly increasing segment (*r* = 0.57) and at steady torque (*r* = 0.52) were not as strong as the relationship previously reported between the *b* terms and muscle cross-sectional area (*r* = 0.67) for the VL by Sontag et al. [27]. It has been reported that muscle thickness may not be as sensitive to total muscle volume as muscle cross-sectional area measurements [51]. Despite the sensor placement and ultrasound imaging site being similar (50% of the distance between the greater trochanter and the lateral condyle of the femur) between the current study and Sontag et al.’s [27], MMG measures the oscillations produced by the entire muscle and not just the motor units underlying the sensor [52]. Another consideration is that non-uniformed hypertrophy has previously been reported for lower limb muscles [53]. Although our subjects were untrained, it is possible that muscle thickness may have varied along the length of the VL, potentially resulting in participants having a greater muscle thickness at a measurement site than that measured in the current study. Nonetheless, the muscle thickness of the VL was positively correlated with the *b* terms during the linearly increasing segment and MMG_RMS_ at steady torque, indicating that individuals with larger muscles exhibited a greater relative increase in MMG_RMS_ with increments in torque and during steady torque production.

Previously, positive relationships have been reported between muscle thickness and pennation angle for lower limb muscles [29,43]. Consequently, it appears that increases in muscle size could be associated with increases in pennation angles [44]. In addition, Maxwell et al. [54] suggested that for muscles of constant length, fiber length, and fiber number, overall muscle size increases require increases in pennation angles and muscle thickness. Thus, it was hypothesized that positive relationships would exist between pennation angle and the *b* terms during the linearly increasing portion of the targeted torque trajectory and MMG_RMS_ at steady torque, which was also supported in the current study. It is suggested that greater pennation angles allow increased contractile proteins within the muscle [44,55,56,57]. Consequently, it is plausible the larger *b* terms and MMG_RMS_ at steady torque may reflect greater overall cross-bridge activity, resulting in an increased amount of oscillations recorded at the surface of the skin. Of note, the relationships between pennation angle and the *b* terms (*p* = 0.77) were stronger than the relationships previously reported (*r* = 0.67) between the *b* terms and muscle cross-sectional area. Conversely, the relationships between pennation angle and MMG_RMS_ at steady torque were not as strong (*r* = 0.571). Therefore, pennation angle appears to have a greater influence on the relative increase in MMG_RMS_ with increments in torque than the amplitude of the MMG signal during constant torque production. Recording MMG_RMS_ up to a high-intensity targeted torque may be the more appropriate muscle action for examining sex-related mechanical behavior and may potentially provide an opportunity for estimating the pennation angle of the VL in sedentary males and females.

Another interesting finding was the sex-related differences in MMG_RMS_ at steady torque. Although previous studies have reported sex-related differences in MMG_RMS_ during linearly increasing muscle actions for upper- [58] and lower-body muscles [27], to our knowledge, this is the first study to report differences between males and females for the VL at a submaximal steady torque/force. Numerous examinations have reported no sex-related differences in MMG_RMS_ of the VL during isokinetic [59,60,61,62,63] and isometric sustained knee extensions [64]. The ability of our study to discern MMG_RMS_ at steady torque in males and females may be attributed to the targeted trajectory we utilized. For example, the linearly increasing segment that preceded the steady torque segment was performed at a slower rate of isometric torque development than traditional step contractions [64] and required less voluntary effort than isokinetic contractions [59,60,61,62,63]. Miller et al. [65] reported that contractions performed at a slower rate compared with a faster rate of isometric torque development resulted in greater recruitment of higher-threshold MUs during contractions at the same relative intensity. Therefore, the activation of a greater percentage of higher-threshold MUs, which are larger for males than females in the VL [28] and possess greater force twitches [66], may be responsible for the sex-related differences in MMG_RMS_ at steady torque.

Another possible explanation for the differences in MMG_RMS_ at steady torque between males and females may be the influence of percent myosin heavy chain expression. It has been reported that percent myosin heavy chain expression provides quantitative information regarding how much of the muscle is occupied by the three major fiber types (i.e., Type I, Type IIA, Type IIX) [67]. Positive relationships have previously been reported between motor unit firing rates and type I percent myosin heavy chain expression [36,68]. In addition, type I fibers possess slower contraction–relaxation rates [66], making motor units that are composed of fibers that express type I characteristics more susceptible to tetanizing [50]. Indeed, once motor units fuse, they are no longer capable of contributing to the amplitude of the MMG signal despite being active [69]. In support, individuals expressing greater type I percent myosin heavy chain expression have exhibited lower MMG_RMS_ values during isometric [23,24,25,26] and dynamic knee extensor testing [70]. Although we did not collect muscle biopsies, the greater mechanical behavior displayed by the males during the linearly increasing segment (*b* terms) and at steady torque (MMG_RMS_) likely suggests percent myosin heavy chain expression differences between the sexes. Lastly, the findings suggest that using a targeted torque/force trajectory that contains a slow linearly increasing and steady segment may be more sensitive to sex-related differences in MMG_RMS_ patterns of response than a rapid contraction.

## 5. Conclusions

In summary, this is the first study to report relationships among pennation angle and muscle thickness with the mechanical behavior of the VL during a linearly increasing and steady muscle action. It has been suggested that larger pennation angles allow increased packing of contractile tissue [7]. This was tentatively supported by the current study as individuals with larger pennation angles displayed greater oscillations at the surface of the skin (MMG_RMS_), and MMG_RMS_ was greater for males, who exhibited significantly greater pennation angles and muscle thicknesses than females. MMG_RMS_ may provide unique insight into muscle architecture and could potentially be used as a measurement tool to inexpensively quantify changes in pennation angle and muscle thickness. Future research should investigate the sensitivity of MMG_RMS_ to potential changes in pennation angle and muscle thickness as a result of training interventions, aging, and/or neuromuscular diseases.

## Data Availability

Please contact the corresponding author for access.
